# Un mode de découverte inhabituel d'hyperparathyroïdie primaire: fractures multiples sur tumeurs brunes secondaires à un adénome parathyroïdien ectopique médiastinal

**DOI:** 10.11604/pamj.2015.22.290.8274

**Published:** 2015-11-24

**Authors:** Asmaa Yassine, Ahmed Anas Guerboub, Adil Arsalane, Abdelhamid Biyi, Souad El Moussaoui, Ghizlaine Belmejdoub

**Affiliations:** 1 Service d'Endocrinologie et Diabétologie, Hôpital Militaire d'Instruction Mohamed V, Rabat, Maroc; 2 Service de Chirurgie Thoracique, Hôpital Militaire d'Instruction Mohamed V, Rabat, Maroc; 3 Service de Médecine Nucléaire, Hôpital Militaire d'Instruction Mohamed V, Rabat, Maroc

**Keywords:** Hyperparathyroïdie primaire, tumeur brune, fractures multiples, adénome parathyroïdien, primary hyperparathyroidism, brown tumor, multiple fractures, parathyroid adenoma

## Abstract

La tumeur brune est une entité clinique bénigne apparaissant comme une manifestation squelettique rare de l'hyperparathyroïdie primaire. Il s'agit d'une cause inhabituelle de fractures pathologiques. Nous rapportons ici le cas clinique d'un patient âgé de 20 ans chez qui l'hyperparathyroïdie primitive a été découverte devant des tumeurs brunes multiples.

## Introduction

L'hyperparathyroïdie constitue actuellement la troisième pathologie endocrinienne après le diabète sucré et la pathologie thyroïdienne [1]. Elle est découverte, dans 75 à 80% des cas, après dosage systématique de la calcémie qui objective l'hypercalcémie qui en découle [1]. Les manifestations squelettiques graves de l'hyperparathyroïdie primitive (ostéite fibrokystique, tumeurs brunes, fractures pathologiques) sont rares de nos jours [2]. Une fracture pathologique révélant ces tumeurs est rare (moins de 2% des cas d'hyperparathyroïdie primaire) ainsi que seulement 2% des cas d'hyperparathyroïdie primaire sont observés avant l’âge de 30 ans [3, 4]. Nous rapportons ici le cas de tumeurs brunes multiples révélées par des fractures pathologiques chez un jeune homme de 20 ans qui présente une hyperparathyroïdie primaire sur un adénome parathyroïdien ectopique médiastinal.

## Patient et observation

**Figure 1:**
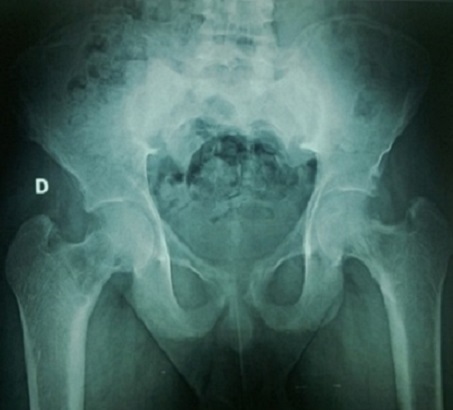
Fracture du col du fémoral bilatérale déplacée et multiples lésions lytiques

**Figure 2:**
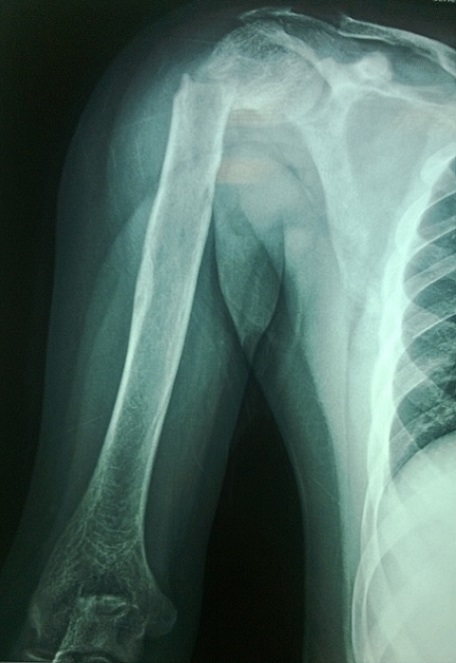
Fracture déplacée du col huméral droit

**Figure 3:**
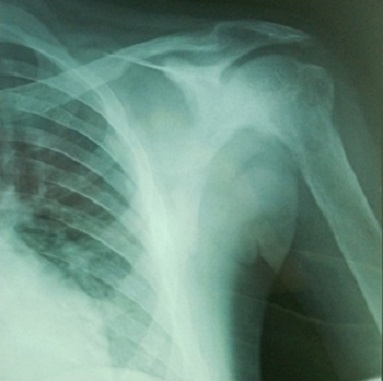
Fracture de l'omoplate gauche et multiples lésions lytiques

**Figure 4:**
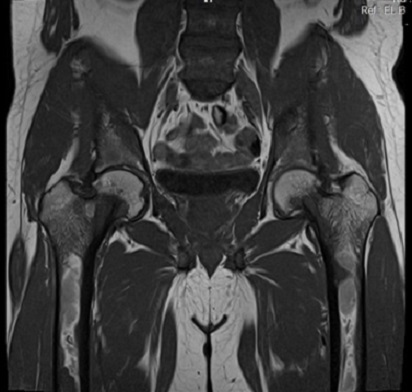
IRM du bassin: multiples lésions hypo intense en T1 qui se rehaussent après injection de gadolinium intéressant les deux ailes iliaques, les deux cols et diaphyses fémoraux

**Figure 5:**
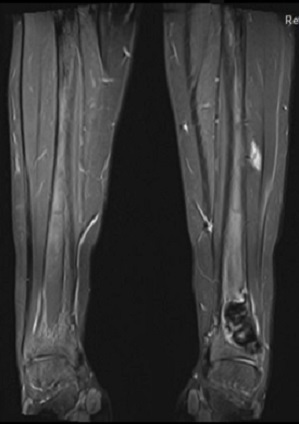
IRM des membres inférieurs: présence d'une formation métaphyso-diaphysaire inférieure du tibia gauche hypo intense T1 et hyper intense T2 se rehaussant de façon hétérogène après injection

**Figure 6:**
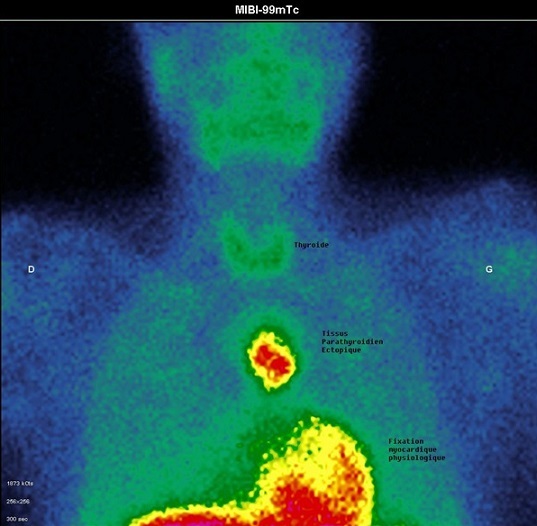
Scintigraphie parathyroidienne au MIBI-Tc 99 m: aspect en faveur de tissu parathyroidien pathologique (flèche blanche) du médiastin supérieur

**Figure 7:**
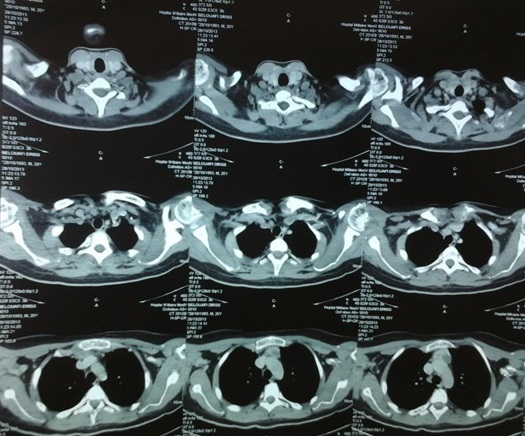
TDM cervico-thoracique: masse médiastinale antéro-supérieur à double composante kystique et charnue

Il s'agit d'un patient âgé de 20 ans, sans antécédents notables, hospitalisé au service d'endocrinologie pour des douleurs osseuses diffuses augmentant progressivement d'intensité depuis 6 mois. L'examen trouve un patient alité multifracturé, hypertendu à 150/90 mm hg, tachycarde à 110 battements/minute sans autres signes associés. Les radiographies standards montrent une déminéralisation osseuse diffuse avec lésions ostéolytiques multiples et plusieurs foyers fracturaires au niveau des deux cols fémoraux (Figure 1), du col huméral droit (Figure 2) et de l'omoplate gauche (Figure 3). L'IRM retrouve de multiples lésions hypointenses en T1 se rehaussant après injections au niveau du bassin, des deux fémurs (Figure 4) et des deux tibias, dont la plus volumineuse mesure 60/31 mm et siégeant au niveau métaphyso-diaphysaire inférieur du tibia gauche (Figure 5). L'analyse anatomopathologique d'une biopsie chirurgicale de la lésion tibiale gauche est non concluante et montre une formation tumorale faite de cellules fusiformes, fibroblastiques associant quelques cellules multinucléées de type ostéoclastes. Devant ce tableau clinico-radiologique un bilan biologique est demandé: Bilan phosphocalcique: calcémie corrigée à 172mg/l, hypophosphatémie à 16 mg/l et une calciurie de 24 heures nomale, une hyperparathormonémie à 4951 pg/ml soit 90 fois la normale, des phosphatases alcalines élevées à 1441 IU/l, une fonction rénale normale, une électrophorèse des protéines plasmatique qui montre un syndrome inflammatoire modéré avec diminution importante de l'albumine. Face à ces données biologiques le diagnostic d'hyperparathyroïdie primaire est retenue et un bilan de localisation de la lésion est effectué notamment l’échographie cervicale qui est normale, la scintigraphie au sestamibi montre un aspect en faveur du tissu parathyroïdien pathologique au niveau du médiastin (Figure 6) et le scanner cervicothoracique (Figure 7) a confirmé les données de la scintigraphie. Vu la sévérité de l'hypercalcémie le patient a bénéficié d'une réhydratation avec perfusion de diurétiques et bisphosphonates, et vu la non amélioration on avait recours à deux séances d'hémodialyse. Après diminution de l'hypercalcémie à 114 mg/l le patient était opéré par thoracotomie avec exérèse de la masse médiastinale. Les suites opératoires ont été marquées par l'installation d'un hungry bone syndrome pour lequel le patient était mis sous supplémentation calcique avec bonne évolution clinicobiologique. L'examen anatomopathologique de la masse médiastinale est en faveur d'un adénome parathyroïdien intrathymique. A noter que la recherche des autres composantes de la néoplasie endocrinienne multiple type 1 est négative.

## Discussion

L'hyperparathyroïdie primitive (HPP) est la conséquence d'une production excessive et inappropriée d'hormone parathyroïdienne ayant pour principale conséquence métabolique une hypercalcémie [4, 5]. Elle est causée par l'existence d'un adénome bénin, unique et sporadique dans 75 à 85% des cas, par une atteinte de plusieurs glandes ou par une hyperplasie dans 15 à 25% des cas. L'association à une néoplasie endocrinienne multiple (NEM I ou II) ou la présence d'un cancer parathyroïdien (< 0,5% des HPP) est très rare [4, 5]. L'HPP s'observe chez l'adulte avec un maximum entre 40 à 50 ans. Seulement 2% des cas peuvent se voir avant 30 ans. L'atteinte féminine semble prédominante [4, 6]. Notre patient est de sexe masculin et de jeune âge (20 ans). Les douleurs osseuses sont vues dans HPP mais se produisent le plus souvent dans l'hyperparathyroïdie secondaire [7] et très rarement une fracture se produit suite à une HPP. L'apparition de fractures pathologiques chez les patients connus pour hyperparathyroïdie est retrouvée chez 15/1 000 patients [8]. La révélation d'une hyperparathyroïdie par une fracture, comme dans notre cas, est très rare [7–9]. Les tumeurs brunes peuvent être totalement asymptomatiques, se manifester par des douleurs osseuses ou encore des fractures pathologiques, comme le cas de notre patient et correspondent sur le plan histologique à une zone d'hyper résorption ostéoclastique contenant un tissu conjonctif inflammatoire hypervasculaire, des cellules géantes, des dépôts d'hémosidérine (d'ou le nom de « tumeurs brunes ») et des zones de tissu ostéoïde qui vont remplacer l'os normal [1, 10]. Les sites classiques d'atteinte sont les os de la face, les côtes, le pelvis, le fémur, les autres os longs et rarement les vertèbres [11] Il est difficile de différencier histologiquement ces tumeurs brunes d'autres lésions à cellules géantes c'est pourquoi l'association de la clinique au bilan biochimique est essentiel au diagnostic. Une calcémie élevée associée à une PTH élevée est très évocatrice du diagnostic d'une HPP [12]. Dans notre cas le diagnostic de l'HPP a été établi devant un dosage de la PTH qui est très élevée. Les radiographies standards ne montraient pas d'autres anomalies osseuses qui auraient pu suggérer une hyperparathyroïdie. La tumeur brune est une entité clinique bénigne apparaissant comme une manifestation squelettique de l´hyperparathyroïdie. Il s´agit d´une cause inhabituelle de fractures pathologiques. La stabilisation du foyer fracturaire et un traitement chirurgical de l´hyperparathyroïdie constituent la clé du traitement de cette entité clinique rare.

## Conclusion

Nous croyons que notre observation contribuerait à la connaissance disponible pour le diagnostic différentiel des fractures pathologiques.
